# Drumine—The New Local Anæsthetic

**Published:** 1887-01

**Authors:** 


ARTICLE V.

### DRUMINE—THE NEW LOCAL ANÆSTHETIC.

The characteristics and properties of the Euphorbiaceæ are so multiple and so different, that it is scarcely a matter of surprise to learn that one of the order has now been shown to yield an anæsthetic. Dr. John Reid, of Port Germein, South Australia, has recently obtained a local anæsthetic from Euphorbia Drummondii, and this drug, for want of a more convenient term, he calls *Drumine*, until chemists give a better name. In his experiments he used the hydrochlorate, *Drumini chloridum*, very soluble in water, and obtained in the following manner: A tincture is made with rectified spirit (though he believes proof spirit would answer especially if acidulated with hydrochloric acid). After standing a few days it is evaporated to get rid of the spirit, ammonia is added in excess, and the solution filtered. The residue, after the ammonia smell is gone, is dissolved in dilute hydrochloric acid, and the filtrate is filtered through animal charcoal to destroy the abundant coloring matter, which is inactive medicinally, but causes a bluish tinge of the skin. The filtrate is evaporated slowly, and leaves the alkaloid. It gives a colorless solution with little taste, almost insoluble in ether, freely soluble in chloroform and water, and depositing from solutions microscopic colorless acicular and stellate crystals, the latter being more numerous in the deposit from aqueous solutions. Roughly speaking the length of the crystals is about one to twenty diameters of a white blood corpuscle (or from 4 to 260 mmm.). Those deposited from hydrochloric acid solution, are filtered through animal charcoal, are circular or boat-shaped at the circumference, and stellate, or perhaps more correctly, discs, as if formed of concentric circles, and with radiating or other fissures. Under high power the acicular crystals are sometimes of a rhomboid shape, and seem less soluble in chloroform; from which it may be suspected that there are two alkaloids, though Dr. Reid was unable to investigate this point.

Dr. Schomburg, of the Adelaide Botanical Gardens, who determined the species of the plant for Dr. Reid, states that many sheep and cattle are annually killed by eating it, and that it is more poisonous according to the quantity of milky juice which it contains. It is said that animals die within twenty-four hours to seven days after eating it, all being paralyzed in the extremities, and some hanging the head as though intoxicated, though the appetite does not seem to be impaired. It is also said that yellow eyes, and even jaundice, occur in some cases. Its most remarkable and valuable effect, however, is that of causing local anæsthesia. When a few drops of a watery solution, estimated at 4 per cent., was dropped into the eye of a cat, the eye was found in a few minutes to be tolerant of contact with the finger, and the orbicularis muscle did not contract firmly as did the other. The pupil was not appreciable dilated. Three grains injected subcutaneously into the back had no other apparent effect than local anæsthesia. When Dr. Reid applied it to his tongue, nostrils and hand very marked anæsthesia was produced; even the sense of taste for quinine was abolished on the side of the tongue to which it was applied. Small doses taken internally seemed to produce no constitutional effects. In a case of sciatica in an old man, in which he had tried iodide of potassium and ammonia, the first hypodermatic injection of 4 minims of a 4 per cent. solution, enabled him to stand and walk in a short time with comparative ease, the pain having disappeared. A second injection, on the following day, acted as well, and at the time of writing the report the pain had not returned. Dr. Reid used it for cases of sprain, and the "speedy effect is probably sufficient to allow us to bid farewell to evaporating and lead lotions. I have seen it work like magic in a boy, (boil?) the dose being the same, and injected over the adductors of the thigh. For tic I have dropped it into the eye and applied locally with success."

In regard to the physiological action of drumine: "Where death arose from an overdose of the drug, paralysis of the extremities occurred. . . . and we may suppose that the posterior cornua of the cord (sensation) are primarily affected, the poison passing to contiguous parts. I am very much inclined to believe that reported cases of paralysis are neither more or less than cases in which movements remain, but the sense relations with the external world exist to a very limited extent, while motion is still possible. . . . It will be seen that strychnia, as an antidote, affects only the motor parts, there is *possibly an antagonism* to a slight extent between chloroform and the drug. . . There seems to be no special action on the pupil, although the cornea is insensitive by local application. In no case have convulsions followed its use. To sum up; cocaine seems to have a mixed action, sensory and motor, to cause preliminary excitment—Drumine is almost a pure sensory (no action on the pupil) paralyzes, without preliminary excitement; can be given with comparatively slight, if any, risk. A fungus is generated in the drug solution after some days. Its uses may be summed up as follows: Nerve troubles of a painful character, not due to a constantly exciting cause which remains potent, operations, irritation, œdema, sprains and such like; but I believe there is a brilliant future for this drug in the domain of cerebral physiology on account of its almost purely topical action. For hydrophobia, and croup with spasms, it would be used fearlessly and applied either to the nostril, by spray, or with a very fine hypodermic needle into the larynx. Those daring spirits who inject antiseptics, *e. g.*, corrosive sublimate to the phthisical lung, will probably, in this drug, find a valuable adjunct. Let us hope, from its causing no preliminary excitment, it may be useful in peritoneal and bowel ailments of a painful nature, whether by hypodermic needle or by the mouth. Writer's cramp and its congeners appear to indicate its use." In cases of poisoning by it Dr. Reid would recommend Epsom or Glauber's salts alone, or combined with tartar emetic in small doses, with plenty of fresh food, in order to eliminate it and act antidotally.

Of course the correct interpretation of facts, on untrodden ground, with a limited supply of a drug, and without such apparatus for manufacture and experimentation as would be desired, is very difficult; and certainly no one would be inclined to quarrel with Dr. Reid for not telling us more about *Drumine*—on the other hand, the world is his debtor, especially that portion of the medical world which is in the habit of making annual addresses, and which has been ringing the changes on cocaine for two years. Let us hope that, so long as they have no new facts to present, they will at least give part of their attention to *Drumine*. Dr. Reid's interesting paper may be found in the *Australasian Medical Gazette* of October 15, 1886.—*Editorial Journal of Am. Med. Association.*

